# Novel Biomarkers for Risk Stratification and Identification of Life-threatening Cardiovascular Disease: Troponin and Beyond

**DOI:** 10.2174/157340312801784943

**Published:** 2012-05

**Authors:** Louai Razzouk, Mario Fusaro, Ricardo Esquitin

**Affiliations:** 1 Division of Cardiology, Department of Medicine- NYU Langone Medical Center; 2 Department of Medicine- NYU Langone Medical Center; 3 Division of Cardiology, Department of Medicine- Beth Israel Deaconess Medical Center

**Keywords:** Biomarkers, risk-ST ratification.

## Abstract

Chest pain and other symptoms that may represent acute coronary syndromes (ACS) are common reasons for emergency department (ED) presentations, accounting for over six million visits annually in the United States [1]. Chest pain is the second most common ED presentation in the United States. Delays in diagnosis and inaccurate risk stratification of chest pain can result in serious morbidity and mortality from ACS, pulmonary embolism (PE), aortic dissection and other serious pathology.

Because of the high morbidity, mortality, and liability issues associated with both recognized and unrecognized cardiovascular pathology, an aggressive approach to the evaluation of this patient group has become the standard of care. Clinical history, physical examination and electrocardiography have a limited diagnostic and prognostic role in the evaluation of possible ACS, PE, and aortic dissection, so clinicians continue to seek more accurate means of risk stratification. Recent advances in diagnostic imaging techniques particularly computed-tomography of the coronary arteries and aorta, have significantly improved our ability to diagnose life-threatening cardiovascular disease.

In an era where health care utilization and cost are major considerations in how disease is managed, it is crucial to risk-stratify patients quickly and efficiently. Historically, biomarkers have played a significant role in the diagnosis and risk stratification of several cardiovascular disease states including myocardial infarction, congestive heart failure, and pulmonary embolus. Multiple biomarkers have shown early promise in answering questions of risk stratification and early diagnosis of cardiovascular pathology however many do not yet have wide clinical availability. The goal of this review will be to discuss these novel biomarkers and describe their potential role in direct patient care.

## I BIOMARKERS CHARACTERISTICS 

Scrutiny of new biomarkers must include validating analytical imprecision and detection limits, calibrator characterization, assay specificity and standardization, pre-analytical issues, and appropriate reference interval studies [2]. An ideal biomarker should aid the clinician in diagnosis, prognosis and treatment. It should be readily available and adequately tested, have established reference value compared to a “gold standard”, have known sensitivity and specificity, a rapid turnaround time and not be costly [3].

## II THE ROLE OF INFLAMMATORY BIOMARKERS

Interest in inflammatory biomarkers is a result of the emerging appreciation of inflammation as a key part of the pathophysiologic sequence in the development of both coronary artery disease (CAD) and ACS. 

### C-Reactive Protein

The role of C-reactive protein (CRP) in CAD has garnered significant attention over the past decade. Used as a marker of systemic inflammation, elevated serum levels of CRP have been associated with a wide range of detrimental outcomes, and are often clinically used to guide risk factors modification of stable coronary artery disease (CAD). After the publication of the JUPITER trial, the cardiology community has been split between the proponents of CRP as a mere serological biomarker, and those that argue for its pathophysiologic role in the development of coronary atherosclerosis [4]. In either case, CRP is a very useful biomarker that correlates with worsened outcomes in ACS. In patients presenting with unstable angina or a myocardial infarction (MI), elevated CRP levels correlated with an increased risk of mortality at 14 days, independent of troponin levels [5, 6]. This mortality risk has been confirmed at a longer follow-up of up to 6 months post presentation to the ED, and was even present in those patients who had a negative troponin at presentation [7, 8]. Elevated CRP levels have not been shown to correlate with significantly worsened outcomes in patients with stable angina, however [9].

The prognostic value of CRP extends beyond presentations of ACS, as it correlates closely with worsened outcomes in patients presenting with an aortic dissection or with an abdominal aortic aneurysm [10, 11]. One of the potential drawbacks of CRP as a diagnostic test therefore is its lack of specificity. In a similar fashion to the erythrocyte sedimentation rate (ESR), it is elevated in many disease states, and therefore it is best used to help establish prognosis after a diagnosis is established. 

### Interleukins

Interleukins (IL) are major cytokines that may have clinical applications in the evaluation of potential ACS. IL-6 is a multifunctional cytokine and it plays a central role in inflammation and tissue injury. It acts as a surrogate marker of vessel wall inflammation that eventually leads to atherosclerotic plaque formation. However, there is limited evidence to evaluate the role of IL-6 as a screening tool for chest pain. 

Ridker and colleagues studied the plasma concentration of IL-6 and the risk of future MI in 202 healthy men during a 6-year follow-up [12]. Median concentrations of IL-6 at baseline were higher among men who subsequently had an MI than among those who did not (1.81 versus 1.46 pg/mL; P=0.002); the risk of future MI increased with increasing levels of baseline IL-6 concentration. Also, Pai and colleagues examined the plasma levels of IL-6, CRP and tumor necrosis factor (TNF)-alpha as markers of risk for coronary artery disease among people who were free of cardiovascular disease at baseline [13]. It was concluded that elevated levels of these inflammatory biomarkers indicated an increased risk of coronary artery disease.

Similar to IL-6, elevated levels of serum IL-18 may be an independent predictor of higher cardiovascular mortality, especially among patients with either unstable or stable angina. There is evidence in animal models that IL-18 is associated with increased progression of atherosclerosis and worsened plaque instability. 

This may explain the results of a recent prospective study of 1229 patients with CAD. Median serum levels of IL-18 were significantly higher among patients who had a fatal cardiovascular event than among those who did not (p<0.0001); the hazard risk ratio remained almost unchanged even after adjusting for potential confounders [14]. Serum IL-18 level was also identified as a strong independent predictor of death from cardiovascular causes in patients with coronary artery disease regardless of the clinical status at admission [15].

Balancing IL-18, Interleukin IL-10 is a cytokine that has anti-inflammatory and immunosuppressant properties. In the CAPTURE (c7E3 AntiPlatelet Therapy in Unstable Refractory angina) study, patients with elevated IL-10 levels had a decreased risk of death or nonfatal MI. 

Not surprisingly then, serum IL-18/IL-10 ratio appears to be an independent predictor of recurrent coronary events during long-term follow-up in patients presenting with ACS. In a study done to assess the long-term prognostic significance of admission interleukin-18 (IL-18)/interleukin-10 (IL-10) ratio for recurrent coronary events during a 1-year follow-up in patients presenting with an ACS, it was concluded that serum IL-18/IL-10 ratio is an independent predictor of recurrent coronary events during long-term follow-up in patients presenting with ACS [16]. The study further supports the hypothesis that the balance between pro-inflammatory and anti-inflammatory cytokines may be an important determinant of patient outcome, suggesting a pathogenic role in plaque progression and instability.

### Lipoprotein-associated Phospholipase A2

Lipoprotein-associated phospholipase A2 (Lp-PLA2), an enzyme mainly produced by monocytes and macrophages, generates potent pro-inflammatory products. The sub-endothelial oxidation of low-density lipoprotein (LDL) is viewed as a highly significant biological process that both initiates and accelerates arterial lesion development. Thus, preventing the generation of these mediators through inhibition of Lp-PLA2 is believed to retard atherosclerosis. Consistent with this notion is the recent observation that plasma levels of Lp-PLA2 represent an independent predictor of coronary heart disease and ischemic stroke. 

In the Rotterdam Study, a population-based follow-up study of 7983 subjects over 55 years old, there was a strong relation between Lp-PLA2 activity and coronary heart disease. Compared with the first quartile of Lp-PLA2 activity, multivariate-adjusted hazard ratios for coronary heart disease and ischemic stroke for the remaining quartiles were significant (P for trend< 0.05) [17]. This study showed that Lp-PLA2 activity is an independent predictor of coronary heart disease in the general population.

A case cohort study of 12,819 apparently healthy middle-aged men and women (the Atherosclerosis Risk in Communities study) examined the relation between Lp-PLA2, CRP, traditional risk factors, and risk for CAD events over a six-year period [18]. Lp-PLA2 and hs-CRP levels were higher in the 608 CAD cases than the 740 non-cases. For individuals with LDL-C lower than 130 mg/dL, those with elevated Lp-PLA2 and hs-CRP levels were at the greatest risk for a CHD event.

### Myeloperoxidase

The leukocyte enzyme myeloperoxidase (MPO) is secreted during acute inflammation and causes oxidation of lipoproteins that is linked with the presence of coronary disease. A case-control study of 158 patients with established CAD and 175 patients without CAD found that MPO levels were significantly greater in patients with CAD than in controls (P<0.001). Elevated levels of both leukocyte- and blood-MPO were associated with the presence of CAD. These findings support a potential role for MPO as an inflammatory marker in CAD and may have implications for early atherosclerosis diagnosis and risk assessment. 

More importantly, high levels of MPO may also predict the presence of acute coronary syndrome in patients with chest pain. In a prospective study conducted in 600 patients presenting with chest pain in the ED, plasma levels of MPO predicted the risk of MI even in patients that were negative for troponin T at baseline [19]. In patients without evidence of myocardial necrosis (those who were negative for troponin T), the baseline myeloperoxidase levels independently predicted the risk of major adverse coronary events at 30 days and at 6 months. This finding highlights the utility of MPO for risk-stratification of potential ACS. Among patients who present with chest pain and do not have biomarkers of cellular injury (e.g. positive troponin), MPO may substitute for stress testing as an additional means of risk stratification.

### Oxidized Low-density Lipoprotein

Atherosclerotic plaque inflammation is critical to the pathophysiology of ACS, and oxidized low-density lipoprotein (oxLDL) is thought to be a key mediator of this process [20-22]. OxLDL is a pro-inflammatory mediator highly correlated with adverse cardiovascular events. By inducing local inflammation in the atherosclerotic plaque, oxLDL leads to endothelial dysfunction, plaque expansion through foam cell formation, plaque destabilization, and ultimately plaque rupture leading to myocardial infarction (MI) [23]. 

The MIRACL study sought to define the relationship between oxidative biomarkers and CVD risk factors (Results from the MIRACL [Myocardial Ischemia Reduction With Aggressive Cholesterol Lowering] trial). The study measured oxLDL at baseline and 16 weeks of treatment with 80 mg Atorvastatin in over 2300 patients with ACS. In these patients, baseline levels of oxidative biomarkers varied according to specific CVD risk factors and were largely independent of inflammatory biomarkers. Atorvastatin administration led to a decrease in total oxidized phospholipids in ACS patients. Future studies are warranted to assess whether decrease in oxLDL levels correspond with improved cardiovascular outcomes.

### Matrix Metalloproteinase

Microscopic study of atherosclerotic plaque (both coronary and aortic) in humans has demonstrated excess matrix degrading activity, suggesting that elevated levels of matrix metalloproteinase (MMP) might act as a surrogate marker of atherosclerotic plaque burden. Clinically however, elevated levels of MMP have demonstrated no utility in evaluation of ACS patients [24], though they may help identify patients presenting with an aortic dissection [25].

## III. MARKERS OF LEFT VENTRICULAR FUNCTION AND NEUROENDOCRINE ACTIVATION

### Brain Natriuretic Peptide

Brain natriuretic peptide (BNP) level measurement was initially studied as a tool for differentiating cardiac from non-cardiac causes of dyspnea. In a landmark 2002 study, Maisel *et al*. demonstrated that the test had over 90% sensitivity for detecting heart failure in patients with acute dyspnea presenting to the ED when compared to a cardiologist who had access to the patient’s entire medical record [26]. The utility of BNP in an ED setting was confirmed in several subsequent studies as well [27]. Based on these studies, a recent consensus algorithm was created in which patients with BNP levels below 100 were determined to have a very low (<2%) chance of heart failure, whereas patients with serum BNP levels above 400 were very likely (>95% chance) to be in heart failure [28]. Of note, BNP levels may be elevated in septic shock and massive pulmonary embolus; therefore caution must be used in its interpretation when these are suspected. 

BNP has also demonstrated some utility as a prognostic marker of CHF; inpatient mortality is related to admission levels of BNP in a linear manner [29,30]. It may also be useful as a prognostic measurement prior to discharge; levels above 350 are highly predictive of short term readmission [31]. 

### Troponin

In recent years, troponin has become the gold standard in detection of MI over its predecessors, CK-MB, CPK and AST. Its efficacy in diagnosis of MI has been well-validated. As test methods improved, the superiority of cTroponin I (cTnI) over T was established and as sensitivity for each assay increased, specificity for acute coronary syndrome has been called more and more into question [32, 33]. A new study by Mills and coauthors compared highly sensitive troponin I to standard troponin I and the effect on lowered thresholds on outcomes [34]. This was a two-part study with validation and implementation phases that followed 2092 patients over one year to measure event-free survival (MI and death) in consecutive patients presenting with suspected ACS. The validation phase of the study measured both standard and high-sensitivity troponin (but only reported troponin I thresholds of 0.20 ng/mL to clinicians) and objectively measured treatments. The validation phase reported the hs-CTNI. In patients with hs-CTNI levels of 0.05-0.19 ng/mL, there was a reduction in recurrent MI and death at one year from 39% to 21%, (OR 0.42, CI 0.24-0.84; P=0.01) between the validation and the implementation arms, respectively. The decrease in primary endpoint was attributed to more aggressive management in that subset of patients. Given that immediate outcomes were not reported, benefit in these patients is likely related to increased secondary prophylaxis in the implementation group and lack of primary prophylaxis in the validation group.

Although the role of troponin in the identification of myocardial infarct has been clearly established, its use in detection and prognostication in the setting of PE is emerging as a potential tool in PE management. In a meta-analysis of 20 studies and 1985 patients, the investigators looked at the predictive value of troponin T and I on patients admitted to the hospital with PE [35]. The primary outcome was death, with secondary outcomes including shock, need of vasopressors, intubation or thrombolysis. In patients with an elevated troponin, there was an overall incidence of death of 19.7% vs. 3.7% in those without an elevated troponin level. Jimenez *et al* published a study not included in the analysis above, and studied this potential in a prospective cohort study of normotensive patients who were confirmed to have PE [36]. Their results revealed a significant association with a right bundle branch block, an S1 Q3 T3 pattern on EKG, as well as sinus tachycardia. There was a non-significant increase in 30-day mortality. In addition, they found a negative predictive value of 93% for troponin I levels <0.1 ng/mL. This lack of association with death was again noted when troponin I was used alone in a study by Bova *et al* [37]. Interestingly, when high-sensitivity cardiac troponin (hs-cTnT) was used, Lankeit *et al* found a two-fold increase in adverse outcome for every standard deviation increase in hs-cTnT [38]. 

## IV SEROLOGICAL MARKERS OF BLOOD VULNERABILITY

### D-dimer

After clot formation, fibrinolysis of said clot leads to formation of fibrin split products, in particular D-dimer. The utility of this protein fragment has been well validated in the past for ruling out PE or DVT in patients with low or medium pretest probability. More recently, the role of D-dimer has been further established in the setting of aortic dissection as well as PE clot burden, location of PE and prognosis.

In consecutive patients presenting to the ED, levels of D-dimer have been significantly associated with prognosis. Klok *et al.* found that in patients with confirmed PE, D-dimer >3000 ng/mL are associated with increased death at 15 days, at 3 months, as well as a more centralized location of PE [39]. A direct correlation between increased D-dimer levels, worsened prognosis and more central clot burden were also noted in several other trials of new PE diagnoses [40, 41].

Interestingly, in hospitalized patients who are already hemodynamically stable, elevated D-dimer in PE does not have any prognostic value up to at least three months after diagnosis [42]. These conflicting data might suggest that less fragile patients have been sub-selected for these studies and that if one does not initially become unstable, they can survive significant clot burden with elevated D-dimer levels in at least the short term.

PE is not the only life-threatening condition that can be ruled out with D-dimer. Emerging evidence suggests D-dimer can also greatly reduce post-test probability of aortic dissection as well. As intima is exposed, coagulation ensues with resultant liberation of fibrin split products. Hazui *et al*. reported that patients presenting to the ED for chest pain with a D-dimer >0.8 g/mL and widened mediastinum had a sensitivity and specificity of 93.1% and 91.8%. [43]. Negative predictive values are also similar to those of PE. In a study by Sodeck *et al*., patients presenting for suspected aortic dissection with D-Dimer values <0.9 ug/mL had a negative predictive value of 92% and for <0.1 umg/mL, 100% [44]. 

### Fibrinogen

As inappropriate hemostasis can lead to coronary thrombosis unexpectedly, elevated levels of certain coagulation factors can be implicated in future thrombotic events. In a meta-analysis by Danesh *et al*., 4018 pts from 18 prospective studies were evaluated for an association between fibrinogen and future vascular events [45]. In patients in the upper third of fibrinogen levels vs. the bottom third, there was a risk ratio of 1.8 for coronary heart disease (MI, CAD, valvular disease) after being controlled for classic risk factors. Patients who presented for ACS and had fibrinogen levels of >3.4 g/L had a mortality rate of >12% after a mean 37 months of follow up [5].

### vWF

As platelet activation is intimately related to thrombosis during ACS, it would stand to reason that markers of platelet activation might be useful in predicting outcomes after ACS. In the past, von Willbebrand’s Factor (vWF) has been studied as a marker of potential treatment selection and therapeutic target. Additionally, its potential has been explored as a prognosticator for adverse outcomes. 

Collet *et al*. evaluated levels of vWF in consecutive patients presenting for ST elevation MI (STEMI) or Q-wave MI at admission and 24 hours later [46]. In this group, vWF levels at 24 hours were significantly elevated in patients who experienced death at 30 days compared to those who did not die in that period. vWF levels and their relation to outcomes in STEMI patients were retrospectively studied in the ENTIRE TIMI 23 trial as well [47]. It was noted that patients with vWF levels in the 75th percentile at 48 hours had a combined incidence of death or MI of 11.2% at 30 days compared with 4.1 in the lower 3 quartiles.

In the ESSENCE trial, vWF was investigated along with several other factors in patients with unstable angina and q-wave infarction [48]. The authors concluded that patients presenting with unstable angina (UA) or non-ST elevation MI (NSTEMI) who experienced the composite endpoint of death, recurrent angina, MI, or revascularization at 14 days had on average double the vWF level as those who did not. It was also noted that enoxaparin significantly reduced the concentration of vWF as compared to unfractionated heparin, suggesting another potential mechanism by which its superiority over unfractionated heparin is achieved. 

### svCAM-1

Similar to vWF, the potential for detection of disease and risk stratification in soluble vascular cell adhesion molecule-1 (svCAM-1) is related to the pathogenesis of thrombosis. Rallidis *et al*. measured levels in patients presenting for severe UA or NSTEMI and demonstrated that sVCAM-1 was significantly elevated at presentation in those who later experienced major adverse cardiac events (death, nonfatal acute myocardial infarction, and recurrence of angina) during their hospitalization, which was independent of CRP level or classic risk factors [49]. There was no correlation with other molecules of this family (sICAM-1, sP-Selectin, sE-selectin) and adverse events. 

### sCD40L

Soluble CD40 ligand (sCD40L) is involved in a variety of processes from atherogenesis to plaque destabilization and can be detected before and during MI. SCD40L is produced in a multitude of cells, most notably in endothelium, activated platelets and activated leukocytes. Although it is not yet a widely available lab test, preliminary evidence indicates potential for treatment selection, risk stratification and prognostication [50]. 

In a trial by Heeschen *et al*., patients with angiographically confirmed coronary artery disease who presented with unstable angina were randomized to treatment with dalteparin or placebo 18-24 hours before angioplasty [51]. The primary outcome was death or MI at 72 hours, 30 days and 6 months. Patients in either group were further subdivided by sCD40L level. For patients with sCD40L levels of > 5.0 ug/L in the placebo group, the rate of primary outcome at 72 hours, 30 days and 6 months was 13.1%, 14.5% and 18.6%, while in patients with sCD40L levels < 5.0 ug/L it was 4.3%, 5.3%, and 7.1%, respectively. Additionally, this study was able to detect patients who might derive benefit from abciximab treatment based on the level of sCD40L. In the second part of the study, it was found that patients with sCD40L levels >5.0 ug/L who received abciximab had a hazard ratio of 0.37 (CI 0.20-0.68). These findings were independent of troponin levels. 

Malarstig *et al*. found similar results in patients presenting for suspected NSTEMI [52]. In this trial sCD40L levels above the median were associated with a 2.5-fold increase in MI. This risk was reduced with use of dalteparin during the 90-day treatment period but became non-significant at the 24-month conclusion. 

Schonbeck *et al*. conducted a prospective, nested case-control study in which they studied 130 women who died of MI, stroke or other cardiovascular cause [53]. After being matched for known cardiovascular risk factors, it was noted that the cases had a significantly elevated sCD40L level compared with controls (2.86 ng/mL versus 2.09 ng/mL). These results were not corroborated in a study by Apple *et al*., in which they sought to find associations with several biomarkers and adverse outcomes during and after acute coronary syndromes [24]. SCD40L was not noted to have a statistically significant association with death during or after MI.

### Growth Factors

Growth factors have been getting significantly more attention in recent years given the importance of angiogenesis in collateralization during progression of atherosclerosis. The most studied factors as pertains to coronary disease are vascular endothelial growth factor (VEGF), placental growth factor (PlGF) and hepatocyte growth factor (HGF). 

VEGF produced in a variety of processes and in the setting of coronary disease has been used to predict future adverse events. Baldus *et al*. found that in patients presenting with ACS, VEGF levels > 300 ug/L were associated with a hazard ratio of 1.87 (CI 1.03-3.51) for MI and death during 6 months of follow up [54]. 

In a paper by Heeschen *et al*., serum was obtained from 547 patients admitted for ACS [55]. The rate of death or MI at 30 days was 14.8% in patients with PlGF levels of > 27.0 ng/L, compared to 4.9% in those with PlGF values <27 ng/L (HR 3.34 CI 1.79-6.24). Similar results were noted in a follow-up of this same cohort by Lenderink *et al*. over a 6-month period, as well as a separate prospective trial by Apple *et al*. (despite the use of a lower PlGF level of 17 ng/L) [24,56]. 

HGF, a potent angiogenic agent related to recovery after MI, may carry prognostic information as well. Heeschen *et al*. found that in patients who presented with unstable angina, HGF levels > 4.7 ug/L were associated with a protective effect at 72 hours, 30 days and 6 months with HR of 0.29 (CI 0.16-0.53), 0.30 (CI 0.18-0.52) and 0.33 (CI 0.21-0.51), respectively [55]. This may potentially signify a patient’s ability to collateralize during progressive coronary stenosis or may demonstrate is protective effect in another fashion.

## V CONCLUSION: HOW SHOULD WE USE ALL THIS INFORMATION?

As the number of cardiovascular biomarkers grows, it is critical to understand each one’s specific strengths and limitations. Furthermore, it is critical that all biomarkers are not used as stand-alone tests. They must be interpreted in their appropriate clinical context and do not replace other parts of the examination such as physical examination or imaging modalities. Despite the large number of existing and novel biomarkers for cardiovascular disease, no one marker has enough sensitivity and specificity to be evaluated in isolation. Therefore a multi-biomarker strategy is likely to be the most useful for clinical decision-making. In fact, most patients with ACS have multiple processes occurring simultaneously and these biomarkers can help detect distinct points in the pathway of development of ACS.
